# Composition Analysis by UPLC-PDA-ESI (−)-HRMS and Antioxidant Activity Using *Saccharomyces cerevisiae* Model of Herbal Teas and Green Teas from Hainan

**DOI:** 10.3390/molecules23102550

**Published:** 2018-10-06

**Authors:** Hua Li, Lanying Wang, Yanping Luo

**Affiliations:** Institute of Tropical Agriculture and Forestry, Hainan University, Haikou, Hainan, 570228, China; hnulihua@163.com (H.L.); daivemuwly@126.com (L.W.)

**Keywords:** UPLC-PDA-ESI-(−)-HRMS, EPR spectroscopy, *Saccharomyces cerevisiae*, antioxidant activity, H_2_O_2_ stress

## Abstract

Different teas from everywhere are very useful and have been extensively studied. We studied the antioxidant activity of herbal teas and green teas from Hainan, *Mallotus oblongifolius Muell.* Arg. (MO), *Ilex kudingcha* C.J. Tseng (KD), *Camellia sinensis var. assamica* (J. W. Mast.) Kitam. Hainan Dayezhong (DY), and *Camellia sinensis* (L.) O. Ktze. (produced from Hainan Baisha (BS)). The total phenol content and total flavonoid content from water extracts, resin extracts and fractions of herbal teas and green teas were compared. Later, eight fractions of herbal teas and green teas were subjected to UPLC-PDA-ESI-(−)-HRMS. We determined 1-diphenyl -2-picryl-hydrazyl radical and hydroxyl free radical scavenging activity by electron paramagnetic resonance spectroscopy. We subjected *Saccharomyces cerevisiae* to hydrogen peroxide, stress and evaluated antioxidant activity of herbal teas and green teas in *cellulo*. The experiment identified more than 14 potential antioxidant compounds from herbal teas and green teas. The herbal teas and green teas had a clearance rate higher than ferulic acid at the same concentrations. MO best reduced intracellular oxidation levels and increased catalase, glutathione reductase activities, glutathione reduced and glutathione oxidized content. KD had the highest cell survival rate and reduced cell lipid peroxidation. DY best improved superoxide dismutase activity and BS was the most active in the halo test. Therefore, we concluded that MO had stronger antioxidant activity than other herbal teas and green teas from Hainan, especially, which reduce *S. cerevisiae* oxidative stress under H_2_O_2_ stress.

## 1. Introduction

Reactive oxygen species (ROS) are commonly produced as by-products of cellular metabolism, including superoxide anions (O_2_^·−^), HO^·^ and H_2_O_2_. Cells naturally have low levels of ROS that play significant roles in stimulating signaling pathways [1,2]. However, excess ROS can arise from stressful environment or changes in oxidative metabolism of the cell, leading to oxidative stress, lipid peroxidation and cell damage [3]. Oxidative stress caused by excessive production of ROS is a key initiator of many chronic diseases [4]. Therefore, minimizing macromolecular damage caused by ROS can be an effective strategy to slow down aging and prevent age-related diseases such as cancer, Alzheimer’s disease and cardiovascular disease. Antioxidants can prevent ROS production [5,6] and impairment of important molecules, thereby protect the body from subsequent oxidative stress and tissue damage [7,8]. Green tea has very strong antioxidant capacity due to its high catechin content, including (−)-catechin gallate (CG), (+)-catechin (C), (−)-epigallocatechin gallate (EGCG), (−)-epicatechin (EC) and (−)-epicatechin gallate (ECG) [9]. Studies have suggested that herbal teas and green teas can have health benefits due to their antioxidant activity. To date, it is known that *Mallotus oblongifolius Muell.* Arg. (MO) has anti-atherosclerotic effects [10]. *Ilex kudingcha* C.J. Tseng (KD) can protect neuronal cells and the vascular system of rats [11]. It is worth mentioning that *Mallotus* species and *Ilex* species are not *Camellia*, but they can be used as herbal teas, because they all have important antioxidant properties [12,13]. *Camellia sinensis* (L.) O. Ktze. (BS) shows high antioxidant activity in vitro [14]. In addition, *Camellia sinensis var. assamica* (J. W. Mast.) Kitam. Hainan Dayezhong (DY) was certified by the National Crop Variety Approval Committee in 1985 [15], but currently there are few studies that investigate its antioxidant activity, in addition, DY and BS are typical green teas. Therefore, in this article, these four different types of tea from Hainan were called ‘herbal teas and green teas’. However, fewer studies have systematically compared different herbal teas and green teas from a single region and systematically studied the mechanism of action of herbal teas and green teas on *Saccharomyces cerevisiae*. Here, we focused on herbal teas and green teas originating from Hainan, China: MO, KD, DY and BS. It is worth mentioning that these four herbal teas and green tea are endemic to Hainan, and are also the novelty of this experiment. However, do these herbal teas and green teas oxidize? The purpose of this experiment is to explore the effects of Hainan herbal teas and green teas on *Saccharomyces cerevisiae*. We aimed to compare the antioxidant and ROS scavenging activities of these herbal teas and green teas in vitro using Electron paramagnetic resonance (EPR) spectroscopy and in *cellulo* using *Saccharomyces cerevisiae.* EPR spectroscopy is a reliable tool in to investigate radical scavenging and it is comparable to other methods, such as UV [16]. EPR spectroscopy has become an indispensable tool for detecting ROS (O_2_^·−^, HO^·^, HO_2_^·^, RO_2_^·^, RO^·^) through spin trapping [17], and we used to evaluate DPPH^·^ and HO^·^ antioxidant activities in the herbal teas and green teas from Hainan in vitro. At the same time, we used ferulic acid as a positive control [18] after reading the references related to tea and free radical determination by EPR. To evaluate the in *cellulo* role of antioxidants from the herbal teas and green teas, which we used *S. cerevisiae*, a useful model to screen for natural antioxidants. The genome of *S. cerevisiae* has been sequenced and well-studied and is suitable for modification, allowing this model system to play an important role in identifying drugs or gene targets for stress [19]. *S. cerevisiae* has a similar antioxidant response as mammals, and it has functional homologues to 30% of genes involved in human diseases. Therefore, we used two strain yeast cells to test the antioxidant capacity of herbal teas and green teas under oxidative stress induced by H_2_O_2_, the most reactive and toxic ROS [20]. We followed a previously published method [21] to induce an imbalance in the redox state of *S. cerevisiae* in *cellulo*. This study was the first to report the active ingredients and antioxidant activity of DY, and further compare different herbal teas and green teas from Hainan using both in vitro and in *cellulo* models.

## 2. Results

### 2.1. Results of Phenolic Acids and Flavonoids From the Herbal Teas and Green Teas

**Figure 1:**
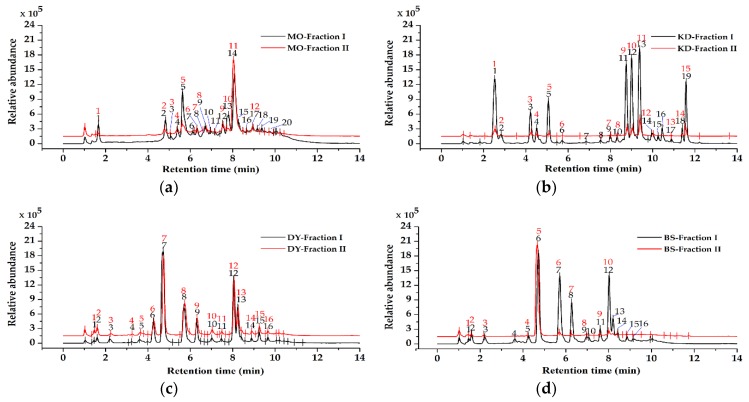
The UPLC-UV-visible chromatogram (270 nm) for the fractions from herbal teas and green teas. (**a**) (**b**) (**c**) (**d**) represent the chromatogram of the two fractions of *Mallotus oblongifolius Muell.* Arg. (MO), *Ilex kudingcha* C.J. Tseng (KD), *Camellia sinensis var. assamica* (J. W. Mast.) Kitam. Hainan Dayezhong (DY) and *Camellia sinensis* (L.) O. Ktze. (BS), respectively.

We separated silica gel chromatography fractions from herbal teas and green teas by UPLC-MS (Figure 1), and the structures of compounds were tentatively identified based on the retention time, UV-visible wavelength maximum, error, MS spectra, standard samples of flavonoids and phenolic acids, and references (Figure S12, Table 1). In MO, it had 14 compounds identified, including ten flavonoids (compound **8**, **9**, **10**, **11**, **12**, **13**, **14**, **15**, **16**, **17**), one phenolic acid (compound **1**), one sterol (compound **7**) and two polysaccharides (compound **4**, **5**). In addition, among the compounds of MO, the top three compounds with the highest content were quercetin (compound **14**), 1-o-galloyl-6-o-luteoyl-a-glucose (compound **5**) and kaempferol-3-o-robinobioside (compound **13**). In KD, it had 15 compounds identified, including eleven phenolic acids (compound **22**, **23**, **24**, **25**, **26**, **27**, **28**, **31**, **32**, **33**, **34**), three flavonoids (compound **29**, **30**, **35**), and one triterpenoid (compound **39**). In addition, the top three compounds of KD with the highest content were 4,5-*o*-dicaffeoylquinic acid (4,5-diCQA) (compound **33**), 3,5-*o*-dicaffeoylquinic acid (3,5-diCQA) (compound **32**) and 3,4-*o*-dicaffeoylquinic acid (3,4-diCQA) (compound **31**). DY contained 16 compounds consisting of two flavonoids (compound **49**, **53**) thirteen phenolic acids (compound **40**, **41**, **42**, **43**, **44**, **45**, **47**, **48**, **50**, **51**, **52**, **54**, **55**), and one alkaloid (compound **46**). Among the compounds of DY, the top three compounds with the highest content were caffeine (compound **46**), ECG (compound **51**) and EC (compound **47**). BS contained 16 compounds, including six flavonoids (compound **64**, **65**, **66**, **69**, **70**, **71**), nine phenolic acids (compound **56**, **57**, **58**, **59**, **60**, **62**, **63**, **67**, **68**) and one alkaloid (compound **61**). In the compounds of BS, the top three compounds with the highest content were caffeine (compound **61**), EGCG (compound **62**) and ECG (compound **67**). In addition, we found that the peaks of fraction II of the four herbal teas and green teas were covered by the peak of fraction I (Figure 1) and we also found that all compounds in the fraction II were found in fraction I.

### 2.2. Total Phenolic and Flavonoid Content

After treatment with AB-8 chromatography, four macroporous resin extracts were obtained (Table 2). Total phenol content (TPC) of the resin extract from MO, KD, DY and BS increased from 353.83 ± 6.49, 186.41 ± 3.47, 326.55 ± 3.21 and 251.26 ± 5.30 mg GAE (gallic acid equivalents)/g to 458.83 ± 5.42, 221.69 ± 0.84, 551.12 ± 5.24 and 428.83 ± 3.74 mg GAE/g, respectively. The increase in TPC was most dramatic in DY-resin. After silica gel chromatography, two fractions were obtained in each herbal tea and green tea. TPC of KD-fraction I, DY-fraction I and BS-fraction I increased to 279.12 ± 1.83, 659.83 ± 1.71, and 526.26 ± 3.00 mg GAE/g, respectively. There was no significant difference between MO-resin and MO-fraction I, it means that MO-fraction I contains almost all of the phenolic acid of MO, and that they can be almost completely separated in the polarity of 50% methanol. TPC of the herbal teas and green teas was higher in both fractions, and fraction I had higher TPC than fraction II, in addition to the two DY fractions. TPC of resin, fraction I and fraction II of DY were the highest compared to other herbal tea and green tea, with TPC of 669.55 ± 4.74mg GAE/g in DY-Fraction II, 659.83 ± 1.71 mg GAE/g in DY-Fraction I, and 551.12 ± 5.24 mg GAE/g in DY-resin. The higher TPC level may be due to the thirteen catechins in DY (Table 1). BS-Fraction I contained nine catechins (Table 1) and its TPC was higher than MO-Fraction I and KD-Fraction I. Following these, the TPC of MO was higher, and there was a significant difference between MO and BS. Moreover, KD had the lowest TPC, and this may be due to the lack of catechin compounds in KD. Total flavonoids content (TFC) was highest in KD, with 742.00 ± 4.65 mg RE (rutin equivalents)/g in water extracts and 786.82 ± 1.56 mg RE/g in resin extracts. DY had the second highest TFC, followed by BS and MO. After silica gel chromatography, DY contained the highest TFC with 974.22 ± 5.31 mg RE/g in DY-fraction I and 960.15 ± 5.87 mg RE/g in DY-Fraction II. This was followed by 858.30 ± 2.79 mg RE/g in KD-fraction I. TFC of BS was higher than MO. Interestingly, TFC was similar across the two extracts and fractions of MO, and fraction I of each herbal tea and green tea had higher TFC than fraction II. In addition, the TPC and TFC between fraction I and II of MO were similar, as well as DY; in contrast, there were significant differences in TPC and TFC between fraction I and II of BS and KD, where fraction I had higher TPC and TFC than fraction II.

### 2.3. Detection of DPPH· and HO· Scavenging Activity by EPR Spectroscope

**Figure 2:**
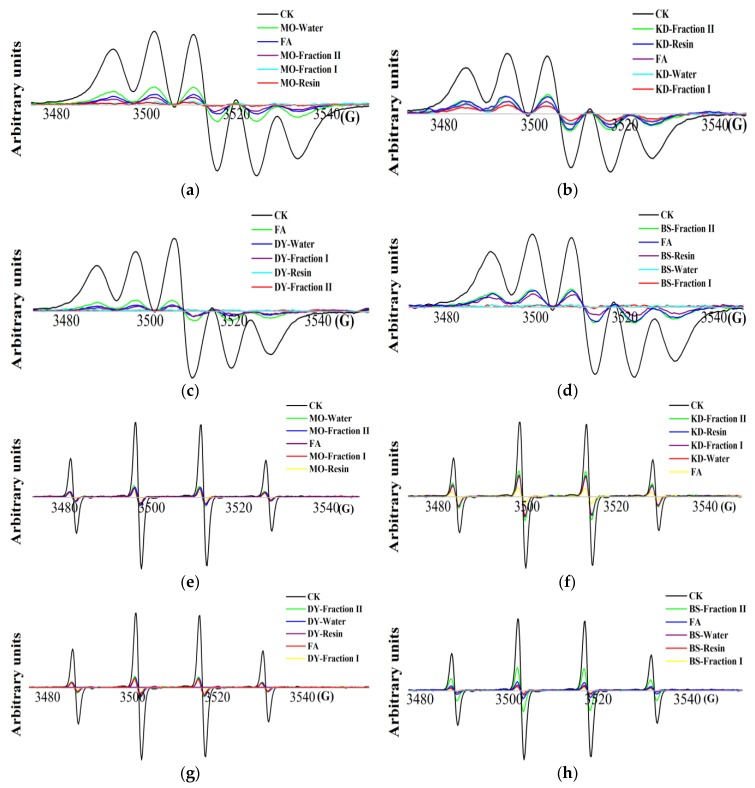
EPR spectra of DPPH^·^ and HO^·^ scavenging: (**a**–**d**) the EPR signal of DPPH^·^ scavenging from four components of one of herbal teas and green teas; (**e**–**h**) the EPR signal of HO^·^ from four components of one of herbal teas and green teas. The abscissa was represented by the G factor, which represents the strength of the magnetic field; the ordinate was expressed in arbitrary units, indicating the relative strength of the signal.

The EPR signal of DPPH^·^ scavenging activity was a 5-fold peak with factor (g) = 2.0043 and hyperfine splitting constants (a) = 8.80 [37]. As the sample concentration increased, the DPPH^·^ peak shape remained unchanged, but the peak height increased, indicating a decrease in radicals (Figure 2a–d). The median inhibitory concentrations (IC_50_) of 16 herbal teas and green teas samples were obtained (Table 3), and herbal teas and green teas extracts with the strongest DPPH^·^ scavenging activity were DY-fraction II, and followed by MO-resin, BS-fraction I, and KD-resin with IC_50_ values of 4.13, 6.54, 12.20 and 18.75 μg/mL, respectively, where scavenging capacity was higher with increasing concentration (Figures S2–S5). IC_50_ of FA was 5.77 μg/mL, which was higher than DY-fraction II, suggesting that the DPPH^·^ scavenging activity of DY-fraction II was higher than FA. The EPR signal of HO^·^ was reflected by 5,5-dimethyl-1-proline-*N*-oxide (DMPO)-OH, a quartet peak with an intensity ratio of 1:2:2:1 with factor (g) = 2.0065 and hyperfine splitting constants (a_N_ = a_H_) = 14.90 G [38]. Similar to DPPH^·^, the signal intensity of DMPO-OH decreased with increasing herbal teas and green teas concentration, where the height of the peak changed, but the shape did not (Figure 2e–h). herbal teas and green teas extracts with strongest HO^·^ scavenging activity were BS-fraction I, and followed by MO-fraction I, DY-fraction II and KD-resin with IC_50_ values of 0.63, 1.21, 1.58 and 4.95 mg/mL, respectively, where scavenging capacity was higher with increasing concentration (Figures S6–S9). Compared to FA (IC_50_ = 1.37 mg/mL), BS-fraction I, BS-resin, BS-water, MO-resin and MO-fraction I showed excellent antioxidant activity levels. Among the 16 kinds of herbal teas and green teas extracts tested in this study, MO-resin, KD-resin, DY-fraction II, and BS-fraction I showed the best scavenging activity, therefore these herbal teas and green teas extracts were selected for further study their antioxidant activity in *S. cerevisiae* cells.

### 2.4. Protection of S. cerevisiae against Oxidative H_2_O_2_ Stress

#### 2.4.1. *S. cerevisiae* Cell Viability

**Figure 3:**
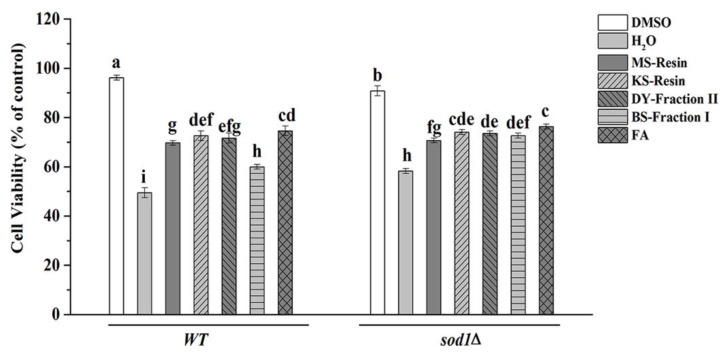
Effect of herbal teas and green teas on survival rates of yeast cells stressed with 2.00 mM H_2_O_2_. The data represented the means ± SD of at least three independent experiments. The capital letters mean statistically different results in two kinds of yeast cells; *p* < 0.05.

We used both wild type (WT) and *sod1Δ* cells to test cell viability (Figure 3). Yeast cell’s viability to H_2_O_2_ stress when treated with MO-resin, KD-resin, DY-fraction II, and BS-fraction I was assessed by measuring survival rate of cells. Cells treated with H_2_O_2_ alone had a low survival rate. Interestingly, cells treated with H_2_O_2_ and herbal tea or green tea extracts showed higher survival. We found that the MO-resin, KD-resin, DY-fraction II, BS-fraction I and FA reliably protected WT and *sod1Δ* cells from oxidative damage. It is noteworthy that KD-resin conferred the strongest ability to increase survival under H_2_O_2_ stress by increasing survival from 49.51% to 72.62% in the WT cells and from 58.27% to 74.09% in the *sod1Δ* cells. Interestingly, there were no differences between KD-resin and DY-fraction II in WT cells and between KD-resin, DY-fraction II and BS-fraction I in *sod1Δ* cells, which showed that the three consistently improved H_2_O_2_ tolerance of *sod1Δ* cells. The survival rate was lower in WT cells treated with MO-resin and BS-fraction I and treated with MO-resin in *sod1Δ* cells, but they were higher than H_2_O_2_ treatment. In addition, we found that *sod1Δ* cells had a higher tolerance to H_2_O_2_ stress than WT cells, and FA improved tolerance of both WT and *sod1Δ* cells.

#### 2.4.2. Cellular Uptake of Herbal Teas and Green Teas Extracts

**Figure 4:**
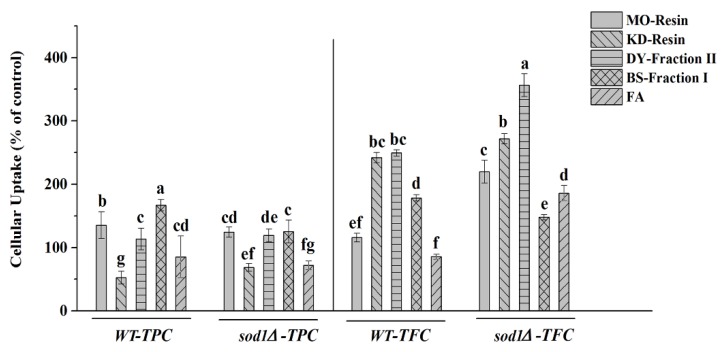
Cellular uptake of herbal teas and green teas components was determined indirectly by measuring the TPC and TFC in the *S. cerevisiae* incubation media supplemented with 200 μL/mL herbal tea or green teas The data represent the means ± SD of at least three independent experiments. The capital letters mean statistically different results in TPC or TFC at *p* < 0.05.

We detected TPC and TFC in the medium after cells were treated for one hour with herbal tea or green tea extract (Figure 4), suggesting that they were not completely taken up by yeast cells. The study found that, the cells absorbed more flavonoids than phenolic acids, and the two cells treated with BS-faction I had the highest absorption of the TPC, where the TPC was 2.16 times higher than the blank treatment, but *sod1Δ* cells treated with BS-faction I showed the lowest absorption of TFC compared to other herbal tea and green tea samples. When treated with DY-fraction II and KD-resin, WT and *sod1Δ* cells had lower TPC and higher TFC absorption. The *sod1Δ* cells absorbed the highest amount of TFC when treated with DY-fraction II, followed by KD-resin, where the TFC was 3.56 times and 2.69 times higher than the blank treatment, respectively. This may be related to the high TFC of DY-fraction II and KD-resin (Table 2). Absorption of TPC and TFC of cells treated with MO-resin was higher than FA. Therefore, the absorption of herbal teas and green teas components by WT and *sod1Δ* cells was higher than FA treatment.

#### 2.4.3. Detection of Lipid Peroxidation

**Figure 5:**
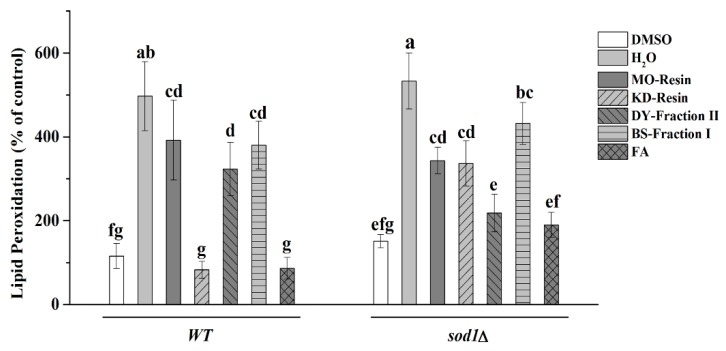
The results of lipid peroxidation levels were expressed as the ratio between treated or not treated with herbal teas or green teas, stressed and non-stressed cells. The data represent the means ± SD of at least three independent experiments. The capital letters mean statistically different results in TPC or TFC at *p* < 0.05.

Oxidative stress could increase lipid peroxidation levels (Figure 5). H_2_O_2_ treatment alone resulted in the highest lipid peroxidation levels, 484.29% in WT cells and 526.10% in *sod1Δ* cells, consistent with its lowest survival rate (Figure 3). But treating cells with herbal teas or green teas extracts could reduce lipid peroxidation levels. In particular, the lipid peroxidation levels of WT cells were reduced from 484.29% to 86.43% under KD-resin treatment. Thus KD-resin showed the strongest anti-lipid peroxidation ability compared with other herbal teas and green teas. DY-fraction II showed the strongest reduction in lipid peroxidation in *sod1Δ* cells, from 526.10% to 219.10% under H_2_O_2_ stress. This was followed by KD-resin 330.40% and MO-resin 340.00%, both of which had anti-lipid peroxidation properties. The effect of KD-resin on WT and DY-fraction II on *sod1Δ* was significant and dramatic compared to H_2_O_2_ treatment alone. Interestingly, the above results of KD-resin in the WT cells and DY-fraction II in the *sod1Δ* cells were not significantly different than treatment with FA. Both MO-resin and BS-fraction I had poor anti-lipid peroxidation ability and there was no significant difference, but both were significantly different from H_2_O_2_ treatment in two yeast cells.

#### 2.4.4. Intracellular Oxidation

**Figure 6:**
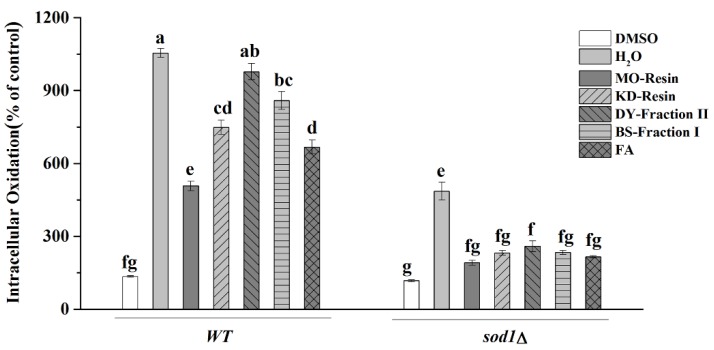
The results of intracellular oxidation were expressed as the ratio between treated or not treated with herbal teas or green teas, stressed and non-stressed cells. The data represent the means ± SD of at least three independent experiments. The capital letters mean statistically different results at *p* < 0.05.

ROS levels increased significantly when WT and *sod1Δ* cells were exposed to H_2_O_2,_ increasing 10-fold for WT cells and 5-fold for *sod1Δ* cells (Figure 6). The increased ROS levels were correlated with the level of lipid peroxidation (Figure 5). Interestingly, the effects of herbal teas and green teas extracts and FA were similar in *sod1Δ* cells, but not in WT cells, where the activity of reduce ROS levels was highest in MO-resin, followed by FA, DY-fraction II, KD-resin, then BS-fraction I. MO-resin suppressed ROS activity the most in H_2_O_2_ treated cells, where ROS levels were reduced by half in WT cells. The activity of reduce ROS levels of BS-fraction I to both yeast cells was lower than that of the other herbal teas and green teas, and this result was consistent with the levels of lipid peroxidation in BS-fraction I treated cells (Figure 5). DY-fraction II and KD-resin significantly could reduce ROS levels, indicating that both could alleviate intracellular oxidation.

#### 2.4.5. Halo Assay

**Figure 7:**
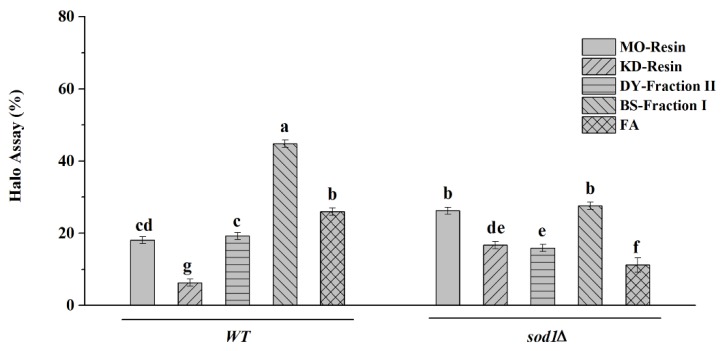
Protective effect of herbal teas and green teas against H_2_O_2_ toxicity. The size of the halo was calculated by the cross method and the results were expressed as the cell-protection rate. The capital letters mean statistically different results at *p* < 0.05.

We next tested whether herbal teas and green teas extracts can reduce H_2_O_2_ toxicity in cells through a Halo assay (Figure 7). H_2_O_2_ solution (2 mM, 2 μL) on the soft agar surface prevents cells from growing in this area. When H_2_O_2_ was placed in an area pretreated with herbal teas or green teas extracts (50 mg/mL, 2 μL), H_2_O_2_ toxicity was alleviated, resulting in a smaller halo (Figure S10). BS-fraction I had the highest cell-protection rate with 44.8% protection in WT cells and 27.6% protection in *sod1Δ* cells. The cell-protection rate of MO-resin was as high as BS-fraction I in the *sod1Δ* cells. In contrast, KD had the lowest cell-protection rate in the WT cells and relatively low protection rate in *sod1Δ* cells. FA protected WT cells more than *sod1Δ* cells, and was the least effective treatment on *sod1Δ* cells. DY-fraction II showed higher effects on WT cells than *sod1Δ* cells.

#### 2.4.6. Determination of *S. cerevisiae* Enzymes Activities

**Figure 8:**
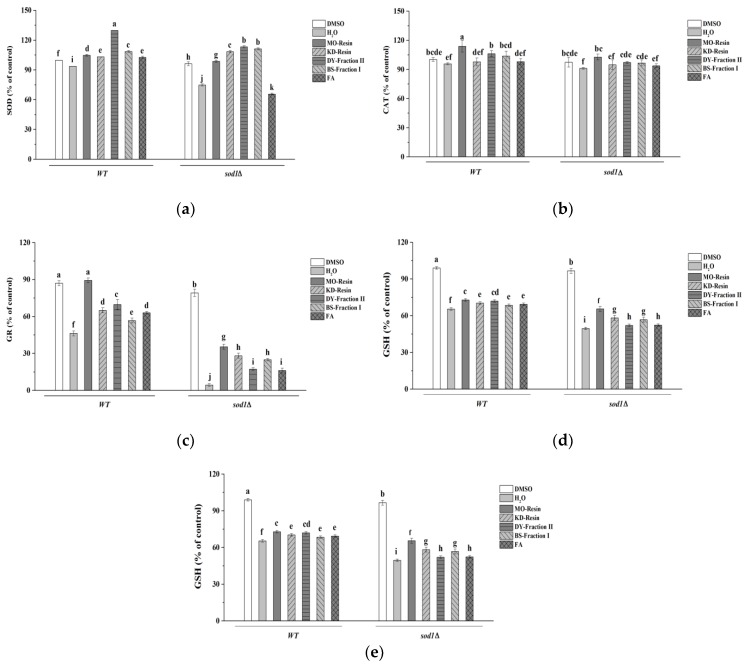
The results of the corresponding enzyme activity of two yeast cells were expressed as the ratio between treated or not treated with herbal teas or green teas. (**a**) SOD activity, (**b**) CAT activity, (**c**) GR activity, (**d**) GSH content and (**e**) GSSG content. The data represent the means ± SD of at least three independent experiments. The capital letters mean statistically different results at *p* < 0.05.

H_2_O_2_ treatment resulted in decreased SOD, CAT, GR, GSH and GSSG enzyme activity (Figure 8). In particular, GR activity dropped from 80.72% to 4.42% in *sod1Δ* cells, GSSG enzyme activity dropped from 96.23% to 16.65% in *sod1Δ* cells and dropped from 97.78% to 22.80% in WT cells. Excitingly, all herbal teas and green teas treatment groups improved the activity of these five preventive enzymes of yeast cells under H_2_O_2_ stress. However, FA did not increase SOD and CAT activity levels in *sod1Δ* cells. Among them, DY-fraction II was the most effective at increasing SOD activity under oxidative stress in WT cells and followed by BS-fraction I, but in *sod1Δ* cells, DY-fraction II and BS-fraction I was no different, which also significantly increased the SOD activity. In CAT activity, MO-resin had the strongest effect, followed by DY-fraction II and BS-fraction I. However, treatment with KD-resin and FA did not result in a significant difference between the levels of CAT activity of cells treated with H_2_O_2_. Furthermore, it was found that MO-resin increased GR activity most, and there was no difference between the MO-resin and the negative control (DMSO), while also increasing the highest activity in GSH and GSSG. At the same time, it was also found that the activity of herbal teas and green teas and FA increased with the same pattern in GR and GSH activities. First, there was no difference between DY and FA, and there was no difference between KD and BS in *sod1Δ* cells, then there was no difference between KD and FA, but there was a significant difference between DY and BS in WT cells. However, GSSG did not have the same results as above. KD, BS and FA had no difference in WT cells, and were lower than DY activity, while in SOD cells, there were no significant differences in KD, DY, and BS, and the activities were higher than FA.

## 3. Discussion

The absorption of flavonoids than phenolic acids played an important role in the antioxidant activity of yeast cells. TPC of water extract from these herbal teas and green teas was not high, it might contain sugar, proteins, and other water-soluble components (Table 2). TPC of four extracts of DY was significantly higher than the other herbal teas and green teas in addition to DY water extract, which may have been caused by the DY water extract without solvent extraction [39]. Herbal teas and green teas from Hainan has higher TFC. A previous study found that TFC of green tea water extract as 81.80 ± 1.43 mg CE/g and resin extract as 105.09 ± 2.24 mg CE/g [40,41]. These values were lower than the TFC of herbal teas and green teas from Hainan, which may be due to the different extraction methods and different species [42]. We used multiple measures to assess the antioxidant activity of the herbal teas and green teas extracts. DPPH^·^ is a very stable free radical, and we detected the EPR signal directly at room temperature before it decayed. EPR spectroscope was used to determine the DPPH^·^ scavenging ability of extracts and fractions of the herbal teas and green teas, representing a total of 16 samples. DY-fraction II had the highest DPPH^·^ clearance effect, and the lowest clearance rate was with KD-resin treatment. The results were consistent with previous finding [43], where DPPH^·^ scavenging rate was positively correlated with TPC. HO^·^ is a short-lived oxygen radical, and its lifetime in aqueous solution is 10^−6^ s. EPR signals for HO^·^ was detected after adding free radical traps to form the relatively stable free-radical adduct DMPO-OH and measured within 3 min [44]. The free radical adducts captured by the DMPO capture were stable compared to the original HO^·^. In this paper, electron-spin capture technology [45] was used to study the scavenging ability of Hainan herbal teas and green teas on HO^·^ produced by Fenton reaction. Further revealing the antioxidant activity of these herbal teas and green teas, we used yeast cells (wild type and mutant) as a model to test the effects of herbal teas and green teas samples on cell antioxidant activity in *cellulo* under oxidative stress induced by H_2_O_2_. H_2_O_2_ treatment resulted in reduced antioxidant activity levels and was toxic to the yeast cells. This result was predictable because H_2_O_2_ treatment results in accumulation of HO^·^, which can destroy organisms [46]. We also used 80% DMSO as a solvent control to treat the cells, and found that it had no effect on yeast cells at the dosages used. Therefore, we found that *sod1Δ* cells had a higher tolerance to H_2_O_2_ stress than WT cells, which may be caused by the higher expression of antioxidants as compensation for the mutation in *sod1Δ* cells [47,48]. We measured the cellular uptake of compounds in the herbal teas and green teas extracts by measuring TPC and TFC of the incubation medium [49,50]. In the halo assay, cells were most protected from H_2_O_2_ when treated with BS-fraction I, which had the highest TPC absorbed by the cells. These results suggest that phenolic acid levels are positively correlated with protecting yeast from oxidative stress and these findings were consistent with previous reports [51]. In addition, we found that 80% DMSO did not result in a halo, indicating that 80% DMSO was not toxic in yeast cells (Figure S11). Membrane proteins are often targets of free radical attacks, which lead to lipid peroxidation, cell leakage and death [52,53]. We analyzed intracellular oxidation and lipid peroxidation in cells treated with herbal teas and green teas extracts to test whether these herbal teas and green teas can protect against the oxidative damage. FA and KD-resin provided protection against lipid peroxidation and intracellular oxidation (Figure 5 and Figure 6), interestingly, the two samples also resulted in high survival rates yeast cells under H_2_O_2_ exposure (Figure 3), and these results were consistent with previous finding [21]. To determine herbal teas and green teas extracts increased tolerance to H_2_O_2_ by reducing the reactive oxidant levels, intracellular oxidation levels were measured by using a probe that produces fluorescent compounds when attacked by ROS [54]. We found that *sod1Δ* cells had lower ROS levels than WT cells, and this was likely caused by the antioxidant system compensating the deficiency in *sod1Δ* cells [55]. There are some antioxidant defense systems in yeast, and the most important is the enzyme defense system. SOD and CAT are critical defense enzymes against ROS. SOD catalyzes the conversion of O_2_^·−^ to H_2_O_2_ to scavenge superoxide free radicals, and CAT catalyzes the conversion of H_2_O_2_ to H_2_O and O_2_. CAT and SOD act in conjunction to clear H_2_O_2_ generated by the SOD catalysis. The concerted catalytic reactions play a vital role in defense against superoxide free radical damage [56]. In our experiments, it was found that all the herbal teas and green teas extracts tested showed increased SOD and CAT activity in yeast cells, especially in *sod1Δ* cells. These results further prove that the herbal teas and green teas extracts influence the antioxidant defense mechanism of *sod1Δ* cells and further increase the activity of scavenging ROS. At the same time, both DY-fraction II and BS-fraction I were significantly increased SOD activity, which suggest that the increase in SOD activity in *sod1Δ* cells may correlate with TPC and TFC absorption from the herbal teas and green teas extracts (Figure 4). However, FA did not improve SOD and CAT activities in *sod1Δ* cells, suggesting that FA was not involved in the defense mechanisms of *sod1Δ* cells, but it plays a role through other pathways for scavenging ROS. The results of this experiment showed that FA increased the enzyme activity of GR and content of GSH and GSSG, which means that FA was used to remove ROS in two cells through glutathione pathway. Similarly, all herbal teas and green teas could improve the activity of GR and content of GSH and GSSG, and MO had the highest activity, which means that these herbal teas and green teas also use this pathway to scavenge ROS and played the antioxidant effect on the cells under H_2_O_2_ stress. This result further explains that there are two ways to remove ROS from cells, one is an enzyme system such as SOD, CAT, and the other is a non-enzymatic system including GSH [57]. GSH is the main intracellular thiol-containing compound that plays a crucial role in the antioxidant defense system. GSH oxidizes to its oxidized form (GSSG), and GSSG can be reduced by GR to regenerate GSH in the presence of NADPH (H^+^) as a cofactor [58]. It was found that the GR, GSH and GSSG of the herbal teas and green teas and FA treatments increased, because when the cells were stressed by ROS, a large amount of GSH was produced in the cells to resist the attack of ROS and form GSSG, and the role of GR is to reduce GSSG to GSH and continue to act on ROS, in a dynamic balance process. In addition, the results show that the activity of MO in CAT, GR, GSH and GSSG were the highest, which were related to the sites of action of their, because their action sites are all on mitochondrial matrix and convert the H_2_O_2_ of the sites into H_2_O and O_2_ [59]. It has been reported that several antioxidants increase some defense enzymes due to through the ERK/Nrf2 pathway and protects against oxidative stress, so this experiment also hypothesized that herbal teas and green teas protects yeast cells from hydrogen peroxide is also related to this pathway [60,61].

## 4. Materials and Methods

### 4.1. Materials

Four types of herbal teas and green teas purchased at Hainan Yesheng Food Co., Ltd. in Haikou, Hainan Province (China) in May 2017; MO was produced in Wanning, Hainan (China) in April 2017; KD was produced in Wuzhishan, Hainan (China) in March 2017; DY was produced in Wuzhishan, Hainan (China) in March 2017 and BS was produced in Baisha, Hainan (China) in April 2017. Wild type *Saccharomyces cerevisiae* cells and its homologous gene-deficient cell *sod1∆* were provided by the College of Biotechnology and Food Science, Tianjin University of Commerce.

### 4.2. Chemicals

Chemicals and kits were purchased from the Beijing Solarbio Company; other reagents were commercially purchased at analytical pure grade.

### 4.3. Preparation of Herbal Teas and Green Teas Extracts

Water extracts of herbal teas and green teas were prepared following published protocols as described below [62]. Herbal teas and green teas leaves were crushed into 60 mesh, and 50 g dry herbal teas and green teas powder was added into 500 mL distilled water, boiled for 45 min, then cooled and filtered. Herbal teas and green teas extraction was repeated three times, and the filtrates were merged, concentrated at 45 °C and dried at −52 °C to yield the dry water extracts.

Resin extracts were generated following published protocols [63]. The AB-8 macroporous resin was rinsed with 95% ethanol to activate it, and then rinsed with distilled water until it was turbid. The 15 mg/mL herbal teas and green teas water extract at pH = 3.0 was poured into a macroporous resin to precipitate the sample at a flow rate of 1 resin volume/2 h. After 1 h, the sugar, organic acids, proteins and other water-soluble components from herbal teas and green teas water extract were eluted with 800 mL water. Finally, water extract was eluted with 80% ethanol at 2 resin volume/h and for 2.5 h. The eluted product was concentrated to yield resin extract of herbal teas and green teas.

200–300 mesh silica gel and 35 cm × 3.0 cm i.d. column was used. The column volume was approximately 10 cm, the column was filled with petroleum ether, and the methanol/chloroform system was used as the elution solvent. The flow rate is approximately one column volume/1 h. Herbal teas and green teas resin extracts were eluted with a 1:1 methanol:chloroform mixture to obtain fraction I, and with 100% methanol to obtain fraction II. In addition, the resin extracts of DY were eluted with 1:4 mixture of methanol:chloroform to yield caffeine in the early stage of the experiment.

### 4.4. Detection of Phenolic Acids and Flavonoids of the Herbal Teas and Green Teas by UPLC-PDA-ESI-(−)-HRMS

The quantitative analysis of herbal teas and green teas was performed using the UPLC-PDA-ESI-(−)-HRMS (LCMS-IT-TOF) instrument [64]. Data acquisition and processing with LCMS Solution version 3.0. An LCMS-IT-TOF (Shimadzu, Kyoto, Japan) system coupled with ultra-high pressure pumps, an Autosampler, a photo-diode array detector (PDA), a column compartment (kept at 45 °C) and a high resolution IT-TOF mass spectrometer with an ESI positive and negative modes were used. Details for the measurement procedures were as follows: HSS-T3 column (Waters): 1.7 μm, 100 mm × 2.1 mm i.d.; a mobile phase: Water/acetic acid (0.1%, *v*/*v*) as solvent A and CH_3_CN as solvent B, at a flow rate of 0.3 mL/min. The gradient program was set as: 10% B (0.01 min), 40% B (5 min), 55% B (8 min), 75% B (10 min), 95% B (12–15 min) and 10% B (15.1 min). The parameters of ESI-MS: Oven Temperature: 45 °C; Nebulizing gas flow and pressure: 1.5L/min and 100 kpa; CDL temperature: 200.0 °C; Detector voltage: 1.7 kV; RP area vacuum: 95.5 Pa; IT area vacuum: 1.8 × 10^−2^ Pa. The MS was calibrated using a mixed calibration solution (trifluoroacetic acid, sodium hydroxide, acetonitrile, methanol, and water in a certain proportion) prepared according to a standard procedure provided by the manufacturer. In the calibration mode, the m/z scan range was 150–2000 nm for post-calibrated. The injection volume was 2 μL for herbal teas and green teas samples and 5 μL for standards. The PDA was set to record peaks at 254, 270 and 360 nm, and UV-visible spectra were recorded from 190 to 800 nm. The peaks were identified by comparing retention time and UV spectrum with standards and references. All samples were dissolved in chromatographic pure methanol and filtered through a 0.22 μm filter before injection.

### 4.5. Determination of TPC

TPC was measured using the Folin-Ciocalteau method [49] with slight modifications. Briefly, 80 μL 1 mg/mL herbal teas and green teas samples (water extracts, resin extracts, and the fractions dissolved respectively in water, 80% ethanol and 100% methanol) and 3.72 mL 2% (*w*/*v*) Na_2_CO_3_ were mixed in a tube, followed by the addition of 200 μL Folin-Ciocalteau’s reagent. The reaction mixture was incubated for 60 min at 40 °C, and the absorption was measured at 760 nm. Experiments were conducted in triplicate, gallic acid was used as standard. The TPC of herbal teas and green teas samples were calculated as GAE with the unit mg GAE/g.

### 4.6. Determination of TFC

TFC was measured following a published protocol [50] with slight modifications. 250 μL of herbal teas and green teas samples, 1.25 mL of distilled water and 75 μL of 5% NaNO_2_ were mixed in a tube, incubated for 6 min, and mixed with 150 μL of 10% AlCl_3_·6H_2_O. After 5 min, 500 μL 1 M NaOH and 275 μL distilled water were added. The mixture’s absorbance was measured at 510 nm immediately after the addition of NaOH and water. The experiment was conducted in triplicate, rutin was used as the standard. The TFC was calculated as RE with the unit mg RE/g.

### 4.7. Detection of DPPH· Scavenging Activity by EPR Spectroscopy

DPPH· scavenging activity was measured using EPR spectroscopy (A300-10/12, Bruker, Beijing, China) following a published protocol [38] with modifications. 100 μL of herbal teas and green teas extracts diluted in methanol (80, 40, 20, 10, 0 μg/mL) was added to 200 μL 0.5 mmol/L DPPH· methanol solution. The reaction was allowed to take place in the dark for 30 min, and the samples were placed in a capillary tube with diameter 0.5 mm, wall thickness 0.1 mm, and length 6 cm. The capillary tube was sealed with wax and placed into the paramagnetic sample tube, which was used for EPR spectroscopy at room temperature. The formula used to calculate the scavenging rate as follows:

DPPH· scavenging rat = (H_0_ − H)/H_0_ × 100%, (1)

where H_0_ was the peak height of the middle peak of the blank control, and H was the peak height in the middle of the sample.

Experimental parameters were as follows: center magnetic field: 3513 G; scan field width: 150 G; modulation frequency: 100 kHz; magnification: 2.00 × 10^4^; modulation amplitude: 1.5 G; scanning times: 1; microwave power: 2.010 mW; time constant: 163.84 ms; and sweep time: 41.943 s.

### 4.8. Detection of HO· Scavenging Activity by EPR Spectroscopy

HO· scavenging activity was measured using EPR spectroscopy (A300-10/12, Bruker) following published protocols [37,38] with modifications. 50 μL of herbal teas and green teas extracts diluted in water (16, 8, 4, 2, 1, 0 mg/mL,) was added to 50 μL 2 mmol/L FeSO_4_, 50 μL distilled water, 50 μL 2 mmol/L DMPO and 50 μL/10 mmol/L DTPA. Afterwards, 50 μL 20 mmol/L H_2_O_2_ was added, and the mixture was placed into a capillary tube. The capillary tube was sealed with wax and put into a paramagnetic sample tube and detected by EPR spectroscopy at room temperature. Measurement process should complete in three minutes after mixing. The formula used to calculate scavenging rate as follows:

HO· scavenging rate = (H_0_ – H)/H_0_ × 100%, (2)

where H_0_ was the peak height of the second peak of the blank control, and H was the peak height of the second peak of the sample.

Experimental parameters were as follows: center magnetic field: 3510 G; scan field width: 100 G; modulation frequency: 100 KHz; magnification: 2.00 × 10^4^; modulation amplitude: 1.0 G; scanning times: 1; microwave power: 2.017 mW; time constant: 163.84 ms; and sweep time: 41.943 s.

### 4.9. Protective Effect of Herbal Teas and Green Teas on S. cerevisiae in Oxidative Stress

We used WT *S. cerevisiae* (yeast) cell BY4741 and its isogenic mutant *sod1Δ* that harbored a knockout in SOD1 of the KanMX4 gene as models for oxidative stress response in these experiments [21]. Activated yeast cells were inoculated in liquid YPD (yeast extract peptone dextrose) medium (100 mL distilled water, 1 g yeast extract, 2 g glucose and 2 g peptone), incubated at 28 °C for 1 day and shaken at 180 rpm, then centrifuged at 3000× *g* for 5 min. The supernatant was removed and yeast cells were washed twice with distilled water, and resuspended in distilled water to measure the absorbance at 600 nm by a UV spectrophotometer, which indicated the concentration of yeast cell suspension was 1 × 10^7^ cells/mL.

#### 4.9.1. Determination of *S. cerevisiae* Cell Viability

Cell tolerance was measured as cell viability [65]. 10 mL cell suspension was mixed with 40 mL liquid YPD medium, then added into 200 μL H_2_O as H_2_O treatment and 200 μL 200 μg/mL herbal teas and green teas extracts as herbal teas and green teas treatment. In addition to this FA dissolved in 80% DMSO was used as positive control, 200 μL 80% DMSO solution was used as the solvent control, and media without the addition of extra compounds was included as a blank control. The mixtures were incubated at 28 °C for 1 h and shaken at 180 rpm. 20 μL H_2_O_2_ (2.0 mM) was added into the above mixtures, shaken at 180 rpm for 1 h. Subsequently, the H_2_O_2_ treated mixtures were diluted 1500 times with sterile water, and 100 μL the dilution solution was placed on a plate containing 2% agar YPD medium (100 mL of distilled water, 1 g of yeast extract, 2 g of glucose, 2 g of peptone and 2 g of agar) in triplicate. The plates were incubated at 28 °C for 72 h, and cell viability was calculated as follows:

Cell viability (%) = (A_0_/A) × 100, (3)

where A_0_ represented the number of cells in H_2_O, 80% DMSO, FA or herbal teas and green teas samples; A represented the number of cells in the blank control.

#### 4.9.2. Determination of Cellular Uptake by Herbal Teas and Green Teas Extracts

Decrease in TPC and TFC of media indicated that the yeast cells took up herbal teas and green teas samples [66]. The samples described above and H_2_O_2_ treated samples were centrifuged at 10,000 g for 5 min, and the supernatant was filtered with an aqueous phase microporous membrane (pore size: 0.45 μm). TPC and TFC of filtrate were measured.

#### 4.9.3. Detection of Lipid Peroxidation

Lipid peroxidation of yeast cells was determined using the TBA method [67]. H_2_O_2_ mixtures described above were centrifuged at 5000× *g* for 5 min to collect yeast cells, and then washed with distilled water. Cells were resuspended in 1 mL 10% TCA (*w*/*v*) containing 5 g glass beads, lysed with three cycles of 20 s agitation by a vortex, then placed on ice for 20 s. The supernatant was centrifuged at 5000× *g* for 5 min. The supernatant of herbal teas and green teas samples had color, therefore the absorbance of the supernatant was measured at 532 nm as the background C_0_, and then the supernatant was treated with 0.2 mL 0.1 M EDTA and 1.2 mL 1% (*w*/*v*) TBA in 0.05 M NaOH in boiling water for 15 min. When the samples were cooled, and the absorbance of the samples were measured at 532 nm as the C, and the absorbance of the samples were calculated as C − C_0_.

#### 4.9.4. Detection of Intracellular Oxidation

Intracellular oxidation was measured using the oxygen sensitive probe DCFH-DA [68]. 10 mL cell suspension was centrifuged at 5000× *g* for 5 min and washed with distilled water. 1 mL 10 μM DCFH-DA solutions were added to the cells and incubated at 28 °C for 15 min to allow for the cells to absorb the probe. After incubation, yeast cells were collected by centrifugation at 5000× *g* for 5 min, and washed with distilled water for 3–5 times to remove the extracellular probe. The yeast cells were inoculated to the liquid YPD medium using the same protocol as above, including blank, H_2_O treatment, DMSO, FA and herbal teas and green teas treatment groups. Samples were incubated at 28 °C with shaking at 180 rpm for 1 h, and 20 μL H_2_O_2_ (2.0 mM) was added to the H_2_O, FA and herbal teas and green teas treatment groups. Samples were further incubated at 28 °C with shaking at 180 rpm for 1 h. The treatment groups were centrifuged at 5000× *g* for 5 min to collect cells, washed with distilled water and resuspended in 500 μL distilled water. Using 3 g glass beads, cells were lysed with three cycles of 1 min agitation by a vortex mixer, then placed on ice for 1 min. The supernatant was centrifuged at 10,000× *g* for 5 min and diluted into 6-fold with distilled water to measure fluorescence at an excitation wavelength of 488 nm and an emission wavelength of 525 nm using a fluorescence microplate reader (Infinite M200 PRO, TECAN, Shanghai, China).

#### 4.9.5. Detection of Halo Assay

The Halo assay was performed on plates containing 0.7% soft agar YPD medium (100 mL distilled water, 1 g yeast extract, 2 g glucose, 2 g peptone and 0.7 g agar) [69]. 10 mL cell suspension (1 × 10^5^ cells/mL) was added into 90 mL 0.7% soft agar YPD medium, which was further divided into five plates. After the YPD medium solidified, three filter papers (0.5 cm) were placed on the medium. 2 μL of the 50mg/mL herbal teas or green teas sample was added to the top filter paper and the right filter paper, respectively; and, 2 μL 80% DMSO was added to the left filter paper. After 1 h, 2 μL H_2_O_2_ solutions were added to the right and the left filter paper. The same method was used with FA and 80% DMSO treatment groups. The medium was incubated at 28 °C for 72 h and the growth zone was measured using the cross method. The halo was calculated as follows:

Cell protection rate (%) = [(B_0_ − 0.5) − (B − 0.5)]/(B_0_ − 0.5)×100, (4)

where B_0_ represented the halo of 80% DMSO + H_2_O_2_, B represented the halo of the H_2_O_2_ + herbal teas or green teas, FA or 80% DMSO, and 0.5 represented the diameter of the filter paper.

#### 4.9.6. Detection of the Enzyme Activity in *S. cerevisiae*

SOD, CAT and GR activity, GSH and GSSG content assay were measured using the commercially available kits (SOD, BC0175; CAT, BC0205; GR, BC1160; GSH, BC1170; GSSG, BC1180).

### 4.10. Statistical Analysis

All experiments involving the yeast cells were repeated at least three times. For each experiment values was shown as the mean ± standard deviation (SD) of duplicate experiments. The differences were considered to be significant when *p* < 0.05. The data and picture analyses were performed using the Statistical Analysis System (SAS 9.1.3), Origin pro 9.0 and ChemDraw Professional 17.0 for Windows.

## 5. Conclusions

We compared the antioxidant activities of herbal teas and green teas from Hainan, and identified that each herbal teas and green teas contained more than 14 antioxidant compounds. DY contained the highest TPC and TFC, and compared to FA, herbal teas and green teas from Hainan showed stronger DPPH^·^ and HO^·^ scavenging activity. In particular, DPPH^·^ scavenging activity was highest in DY-fraction II, followed by MO-resin, BS-fraction I, and the KD-resin. HO^·^ scavenging activity was highest in BS-fraction I, followed by MO-fraction I, DY-fraction II and KD-resin. Furthermore, these herbal teas and green teas alleviated oxidative stress in yeast cells, and MO-resin had the strongest ability to reduce intracellular oxidation levels, and increase CAT, GR activities, GSH and GSSG content. KD-resin improved survival rate and lipid peroxidation under oxidative stress the most. DY-fraction I and BS-fraction I had the strongest impact on improving SOD activity and BS-fraction I was the most active in the halo test. The results support that herbal teas and green teas showed different antioxidant activity caused by the different composition of the herbal teas and green teas, and the initial conclusion was that MO had stronger antioxidant activity than other herbal teas and green teas from Hainan. A comparison of antioxidant properties of herbal teas and green teas from Hainan revealed potential antioxidant compounds, and emphasized the mechanism of action in yeast cells against oxidative stress. Future studies will be beneficial to providing scientific evidence for the utilization of these herbal teas and green teas as antioxidants.

## Figures and Tables

**Table 1:** UPLC-PDA-ESI (−) -HRMS date and putative identification of flavonoids and phenolic acid derivatives from herbal teas and green teas.

**Compounds of MO**	**T_R_** **(min)**	**%RSD**	**[M − H]^−^** **(*m*/*z*)**	**Error** **(ppm)**	**Max** **(nm)**	**Formula**	**Tentatively Identified Compound**
**1**	1.657	0.277	169.0141	0.592	240 271	C_7_H_6_O_5_	Gallic acid [22]
**2**	4.821	0.243	583.1126	Unknown	276 352	Unknown	Unknown
**3**	5.079	0.262	583.1138	Unknown	208 276	Unknown	Unknown
**4**	5.395	0.266	484.0755^a^	5.164	281 341	C_20_H_20_O_14_	1,6-Digalloyl glucose [23]
**5**	5.629	0.295	633.0718	2.369	223 267	C_27_H_22_O_18_	1-*O*-galloyl-6-*O*-luteoyl-α-glucose [24]
**6**	6.122	0.239	388.2395^a^	Unknown	246 269	Unknown	Unknown
**7**	6.314	0.247	415.2388^a^	1.440	254 279	C_29_H_50_O	β-sitosterol [25]
**8**	6.645	0.278	275.0263	2.545	208 263	C_13_H_8_O_7_	3,4,8,9,10-pentahydroxy-dibenzo[*b*,*d*]pyran-6-one [22]
**9**	6.699	0.287	611.1583^a^	3.927	273 208	C_27_H_30_O_16_	kaempferol-3,7-di-*O*-β-d-glucopyranoside [26]
**10**	6.949	0.273	305.0678	3.606	276 208	C_15_H_14_O_7_	(−)-Gallocatechin (GC) [27,28]
**11**	7.154	0.189	385.0843	5.713	271 208	C_19_H_30_O_8_	(6*S*,9*R*)-roseoside [29]
**12**	7.53	0.241	771.197	2.464	278 211	C_33_H_40_O_21_	Quercetin-3-*O*-rutinoside-7-*O*-glucoside [30]
**13**	7.801	0.226	593.1513	0.169	270 214	C_27_H_30_O_15_	Kaempferol-3-*O*-robinobioside [31]
**14**	8.073	0.183	301.0378	7.972	246 217	C_15_H_10_O_7_	Quercetin
**15**	8.274	0.131	431.0985	0.232	255 211	C_21_H_20_O_10_	Vitexin
**16**	8.659	0.134	593.1507	0.843	266 208	C_27_H_30_O_15_	Kaempferol-3-*O*-rutinoside [31]
**17**	8.952	0.118	584.2413^b^	5.341	248 268	C_26_H_34_O_12_	Apigenin [22]
**18**	9.160	0.100	477.0520	Unknown	273 208	Unknown	Unknown
**19**	9.368	0.098	482.0433	Unknown	275 208	Unknown	Unknown
**20**	9.716	0.080	485.0407	Unknown	228 210	Unknown	Unknown
**Compounds of KD**	**T_R_** **(min)**	**%RSD**	**[M − H]^−^** **(*m*/*z*)**	**Error** **(ppm)**	**Max** **(nm)**	**Formula**	**Tentatively Identified Compound**
**21**	2.521	1.666	360.1128	Unknown	253 326	Unknown	Unknown
**22**	2.82	1.534	353.0885	1.983	218 324	C_16_H_18_O_9_	3-CQA [32]
**23**	4.213	1.178	353.0908	8.496	215 322	C_16_H_18_O_9_	5-CQA [32]
**24**	4.507	1.162	353.0885	1.983	218 324	C_16_H_18_O_9_	4-CQA [32]
**25**	5.058	1.026	179.0365	8.378	217 321	C_9_H_8_O_4_	Caffeic acid [32]
**26**	5.707	0.436	337.0928	0.297	223 293	C_16_H_18_O_8_	4-*O*-*p*-coumaroylquinic acid [33]
**27**	6.84	0.847	367.1049	3.012	221 324	C_17_H_20_O_9_	3-FQA [32]
**28**	7.525	0.797	367.1040	3.584	220 326	C_17_H_20_O_9_	5-FQA [32]
**29**	7.99	0.620	609.1508	7.716	203 255	C_27_H_30_O_16_	Rutin [32]
**30**	8.309	0.514	463.0901	4.103	203 255	C_21_H_20_O_12_	Quercetin-3-*O*-galactoside [32]
**31**	8.743	0.370	515.1203	1.553	224 279	C_25_H_24_O_12_	3,4-diCQA [32]
**32**	8.999	0.321	515.1201	1.165	224 277	C_25_H_24_O_12_	3,5-diCQA [32]
**33**	9.376	0.227	515.1204	1.747	225 275	C_25_H_24_O_12_	4,5-diCQA [32]
**34**	9.95	0.155	453.3355	4.191	218 324	C_30_H_46_O_3_	Betulonic acid [32]
**35**	10.253	0.124	529.1347	0.945	221 326	C_26_H_26_O_12_	Macroanthoin G [32]
**36**	10.444	0.105	529.1336	3.024	219 330	C_26_H_26_O_12_	Unknown
**37**	10.88	0.090	543.1523	Unknown	221 326	Unknown	Unknown
**38**	11.395	0.071	647.3196^a^	Unknown	260 326	Unknown	Unknown
**39**	11.574	0.065	469.3701^a^	8.496	251 326	C_30_H_44_O_4_	a-Kudinlactone [32]
**Compounds of DY**	**T_R_** **(min)**	**%RSD**	**[M − H]^−^** **(*m*/*z*)**	**Error** **(ppm)**	**Max** **(nm)**	**Formula**	**Tentatively Identified Compound**
**40**	1.452	0.332	343.0685	4.081	203 270	C_14_H_16_O_10_	5-GQA [27]
**41**	1.596	0.339	169.0149	4.142	215 271	C_7_H_6_O_5_	Gallic acid [22]
**42**	2.194	0.609	305.067	0.983	204 271	C_15_H_14_O_7_	(−)-Gallocatechin (GC) [27,28]
**43**	3.226	0.686	353.0888	2.832	206 274	C_16_H_18_O_9_	3-CQA [32]
**44**	3.605	0.452	305.0671	1.311	204 270	C_15_H_14_O_7_	(−)-Epigallocatechin(EGC)
**45**	4.249	0.433	289.0711	2.422	204 217	C_15_H_14_O_6_	(+)-Catechin (C)
**46**	4.707	0.304	195.0727^a^	2.051	224 273	C_8_H_10_N_4_O_2_	Caffeine
**47**	5.695	0.216	289.0733	5.189	220 277	C_15_H_14_O_6_	(−)-Epicatechin(EC)
**48**	6.289	0.270	457.0771	1.094	204 274	C_22_H_18_O_11_	(−)-Epigallocatechin-3-gallate (EGCG)
**49**	7.01	0.466	563.1143	9.234	200 272	C_29_H_24_O_12_	Theaflavin [34]
**50**	7.458	0.239	471.3439	8.698	206 275	C_30_H_48_O_4_	Pomolic acid [35]
**51**	8.042	0.195	441.0842	3.401	219 275	C_22_H_18_O_10_	(−)-Epicatechin-3-gallate (ECG)
**52**	8.23	0.195	441.0831	0.907	204 276	C_22_H_18_O_10_	(−)-Catechin-3-gallate (CG)
**53**	8.88	0.322	593.1499	2.192	198 266	C_27_H_30_O_15_	Kaempferol-3-O-rutinoside [31]
**54**	9.244	0.246	455.0978	1.318	203 278	C_23_H_20_O_10_	(−)-Catechin-3-*O*-(4-*O*-methyl) gallate [36]
**55**	9.655	0.242	455.0979	1.099	205 278	C_23_H_20_O_10_	(−)-Epicatechin-3-*O*-(4-*O*-methyl) gallate [36]
**Compounds of BS**	**T_R_** **(min)**	**%RSD**	**[M − H]^−^** **(*m*/*z*)**	**Error** **(ppm)**	**M** **ax** **(nm)**	**Formula**	**Tentatively Identified Compound**
**56**	1.458	0.439	343.0645	7.579	213 273	C_14_H_16_O_10_	5-GQA [27]
**57**	1.595	0.374	343.067	0.291	214 271	C_14_H_16_O_10_	3-GQA [27]
**58**	2.202	0.644	305.0652	4.917	203 272	C_15_H_14_O_7_	(−)-Gallocatechin (GC) [27,28]
**59**	3.616	0.545	305.0663	1.311	204 270	C_15_H_14_O_7_	(−)-Epigallocatechin (EGC)
**60**	4.255	0.457	289.0729	3.805	204 280	C_15_H_14_O_6_	(+)-Catechin (C)
**61**	4.718	0.441	195.0725^a^	3.076	222 273	C_8_H_10_N_4_O_2_	Caffeine
**62**	5.712	0.384	457.0771	1.094	216 274	C_22_H_18_O_11_	(−)-Epigallocatechin-3-gallate (EGCG)
**63**	6.269	0.332	457.0763	2.844	218 274	C_22_H_18_O_11_	(−)-Gallocatechin-3-gallate (GCG)
**64**	6.971	0.186	563.1193	0.355	203 271	C_29_H_24_O_12_	Theaflavin [34]
**65**	7.072	0.291	479.0838	1.461	205 265	C_21_H_20_O_13_	Myricetin 3-*O*-β-l-galactopyranoside [35]
**66**	7.597	0.228	771.2002	1.686	203 256	C_33_H_40_O_21_	Quercetin-3-*O*-rutinoside-7-*O*-glucoside [30]
**67**	8.018	0.211	441.0829	0.453	220 274	C_22_H_18_O_10_	(−)-Epicatechin-3-gallate (ECG)
**68**	8.199	0.200	441.0834	1.587	204 275	C_22_H_18_O_10_	(−)-Catechin-3-gallate (CG)
**69**	8.418	0.180	463.0884	0.432	200 266	C_21_H_20_O_12_	Quercetin-3-*O*-galactoside [32]
**70**	8.849	0.120	593.1534	3.709	199 266	C_27_H_30_O_15_	Kaempferol-3-*O*-rutinoside [31]
**71**	9.134	0.103	447.0925	1.789	203 267	C_21_H_20_O_11_	Kaempferol-3-*O*-glucoside [27]

Note: Compounds without reference markers are judged based on retention time and molecular weight of standard samples. ^a^ [M + H]^+^; ^b^ [M + Na]^+^.

**Table 2:** TPC, TFC of extracts and fractions from herbal teas and green teas.

Varieties	MO		KD		DY		BS	
	TPC (mg GAE/g)	TFC (mg RE/g)	TPC (mg GAE/g)	TFC (mg RE/g)	TPC (mg GAE/g)	TFC (mg RE/g)	TPC (mg GAE/g)	TFC (mg RE/g)
Water extract	353.83 ± 6.49b *	215.36 ± 7.00a	186.41 ± 3.47c	742.00 ± 4.65b *	326.55 ± 3.21c *	556.82 ± 26.48c	251.26 ± 5.30c	298.67 ± 6.43c
Resin extract	458.83 ± 5.42a	215.70 ± 2.92a	221.69 ± 0.84b	786.82 ± 1.56ab *	551.12 ± 5.24b *	782.37 ± 9.79b *	428.83 ± 3.74b	499.41 ± 0.76b
Fraction I	449.26 ± 7.49a	288.30 ± 0.31a	279.12 ± 1.83a	858.30 ± 2.79a *	659.83 ± 1.71a *	974.22 ± 5.31a *	526.26 ± 3.00a	602.00 ± 2.41a
Fraction II	433.98 ± 3.78a	237.56 ± 6.83a	158.12 ± 4.35d	446.82 ± 2.01c	669.55 ± 4.74a *	960.15 ± 5.87a *	199.83 ± 4.39d	204.59 ± 7.91d

Notes: Data were expressed as mean ± standard deviation (n = 3); values marked with the different letter within the same column were significantly different (*p* < 0.05) among different extracts and fractions. Values marked by an asterisk were significantly different (*p* < 0.05) between the four varieties in the same extracts or fractions, TPC and TFC were compared separately. All data were based on dry weight basis.

**Table 3:** The half Inhibitory concentration (IC_50_) of DPPH^·^ and HO^·^ radical for herbal teas and green teas.

Components	IC_50_ (μg/mL) DPPH^·^ Scavenging Activity	IC_50_ (mg/mL) HO^·^ Scavenging Activity
MO-water	13.93 ± 0.60 de	1.97 ± 0.05 de
MO-resin	6.54 ± 0.71 fg	1.30 ± 0.17 efg
MO-fraction I	8.33 ± 0.45 f	1.21 ± 0.46 efg
MO-fraction II	8.22 ± 0.98 f	1.62 ± 0.30 def
KD-water	35.04 ± 0.40 b	5.16 ± 0.88 c
KD-resin	18.75 ± 1.30 c	4.95 ± 0.41 c
KD-fraction I	20.26 ± 0.63 c	6.60 ± 0.87 b
KD-fraction II	47.85 ± 1.93 a	8.11 ± 0.33 a
DY-water	4.71 ± 0.15 gh	2.15 ± 0.13 d
DY-resin	4.57 ± 0.09 gh	1.81 ± 0.40 def
DY-fraction I	12.25 ± 1.76 e	1.58 ± 0.24 def
DY-fraction II	4.13 ± 0.70 h	1.44 ± 0.08 def
BS-water	15.48 ± 1.48 d	1.31 ± 0.36 efg
BS-resin	15.93 ± 0.96 d	1.11 ± 0.05 fg
BS-fraction I	12.20 ± 1.55 e	0.63 ± 0.17 g
BS-fraction II	19.74 ± 1.95 c	1.58 ± 0.26 def
FA	5.77 ± 1.23 gh	1.37 ± 0.13 ef

Notes: Data were expressed as mean ± standard deviation (n = 3); values marked by the different lowercase and uppercase letters within the same column were significantly different (*p* < 0.05) among different extracts and fractions.

## Supplementary Materials

The following are available online, Figures S1–S12.

## Abbreviations

MO	*Mallotus oblongifolius Muell.* Arg.
KD	*Ilex kudingcha* C.J. Tseng
DY	*Camellia sinensis var. assamica* (J. W. Mast.) Kitam. Hainan Dayezhong.
BS	*Camellia sinensis (L.) O.* Ktze. (produced from Hainan Baisha (BS))
TPC	total phenol content
TFC	total flavonoids content
UPLC-PDA-ESI-(−)-HRMS^n^	ultra-high pressure liquid chromatography and electrospray ionization coupled with high-resolution mass spectrometry
DPPH·	1-diphenyl-2-picryl-hydrazyl radical
HO·	hydroxyl free radical
WT	wild-type
*sod1Δ*	isogenic mutant yeast cell, harboring the *SOD1* genes knocked out by the KanMX4 gene
YPD	yeast extract peptone dextrose
H_2_O_2_	hydrogen peroxide
DMSO	dimethyl sulfoxide
FA	ferulic acid
CAT	catalase
GR	glutathione reductase
SOD	superoxide dismutase
GSH	glutathione reduced
GSSG	glutathione oxidized
NADPH	nicotinamide adenine dinucleotide phosphate
ROS	reactive oxygen species
O_2_^.−^	superoxide anions
C	(+)-catechin
CG	(−)-catechin gallate
EC	(−)-epicatechin
EGC	(−)-epigallocatechin
ECG	(−)-epicatechin gallate
GCG	(−)-gallocatechin gallate
EGCG	(−)-epigallocatechin gallate
EPR	electron paramagnetic resonance
GAE	gallic acid equivalents
RE	rutin equivalents
DTPA	diethylenetriamine pentaacetic acid
DMPO	5,5-dimethyl-1-proline-N-oxide
TCA	trichloroacetic
TBA	thiobarbituric acid
DCFH-DA	2′7′-dichlorofluorescein diacetate
EDTA	ethylenediaminetetraacetic acid
PDA	photo-diode array detector
i.d.	Inner diameter
LCMS-IT-TOF	liquid chromatography mass spectrometry ion trap time of flight
