# Supplementary material for: Composition Analysis by UPLC-PDA-ESI (−)-HRMS and Antioxidant Activity Using Saccharomyces cerevisiae Model of Herbal Teas and Green Teas from Hainan
Source: Molecules. 2018 Oct 6;23(10):2550. doi: 10.3390/molecules23102550 (PMC6222971; doi:10.3390/molecules23102550)
Supplement: Supplementary file 1 [file molecules-23-02550-s001.pdf]

Composition Analysis By *UPLC-PDA-ESI (-) -HRMS* and Antioxidant Activity using *Saccharomyces cerevisiae* Model of Herbal Teas and Green Teas from Hainan

Hua Li, Lanying Wang and Yanping Luo*

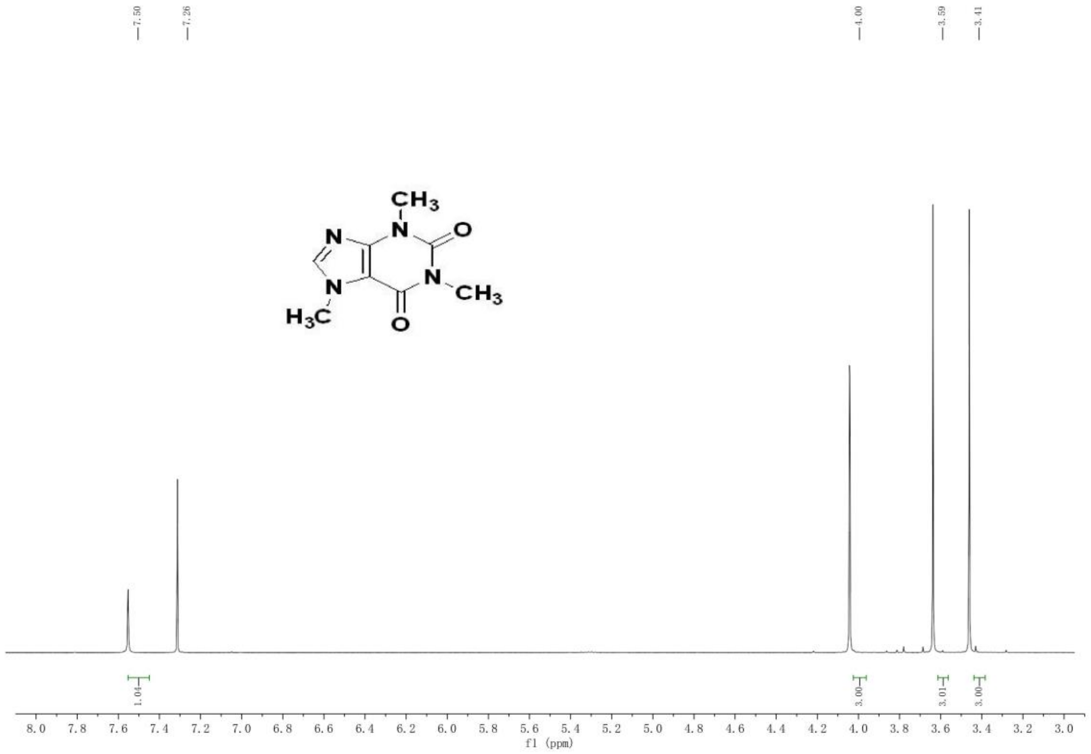

Figure S1. ^1H NMR spectra of caffeine isolated from DY

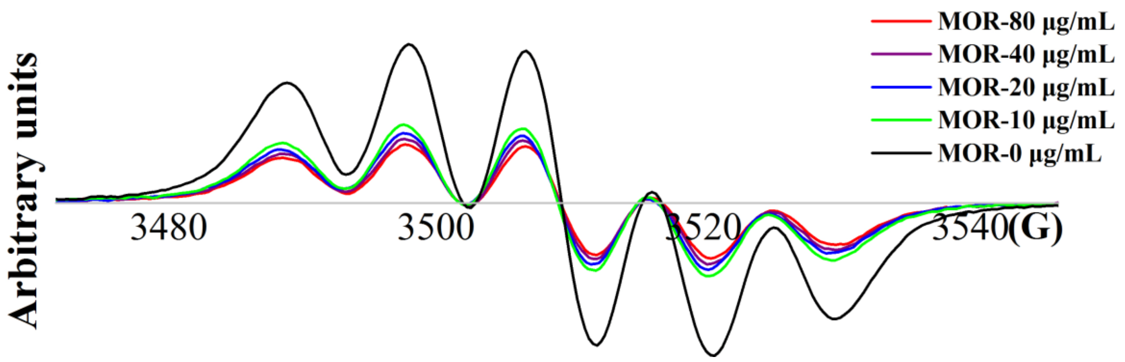

Figure S2. EPR signal strength of DPPH• at four concentrations of MO-Resin

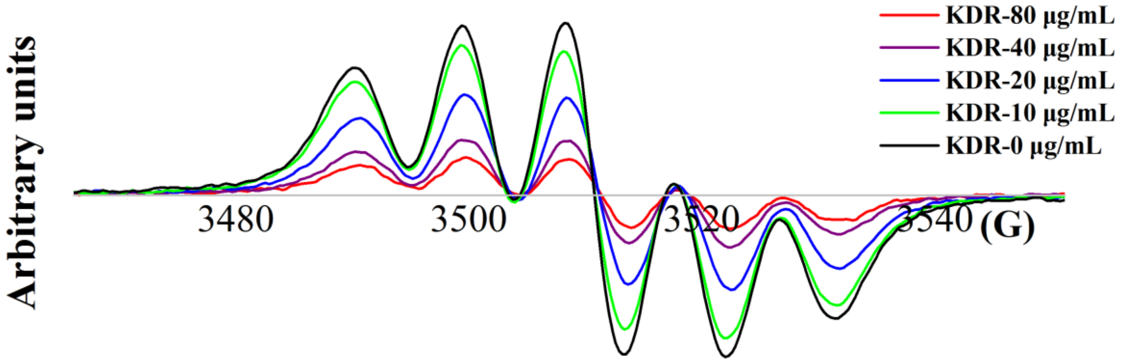

Figure S3. EPR signal strength of DPPH• at four concentrations of KD-Resin

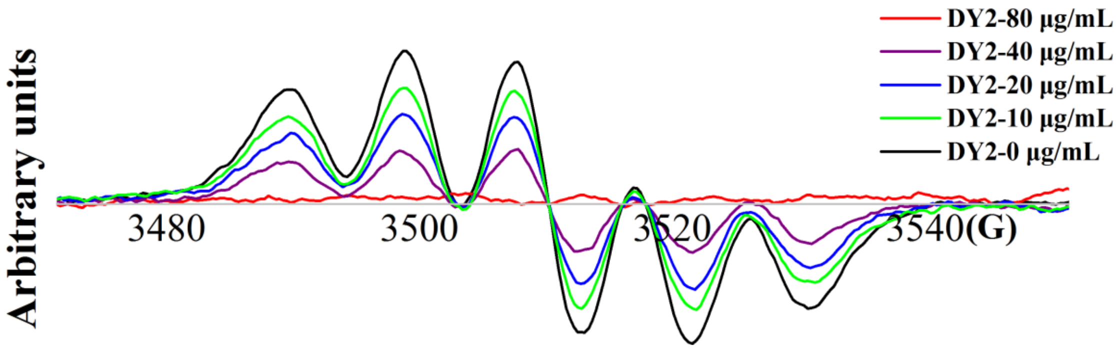

Figure S4. EPR signal strength of DPPH• at four concentrations of DY-Fraction II

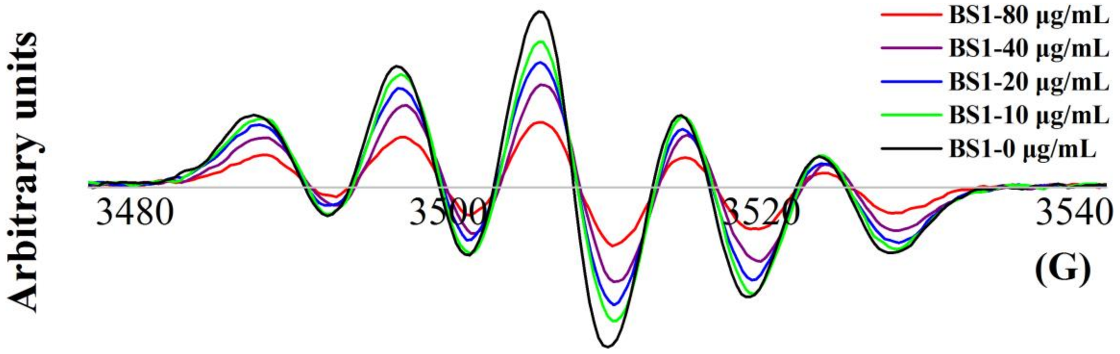

Figure S5. EPR signal strength of DPPH•at four concentrations of BS-Fraction I

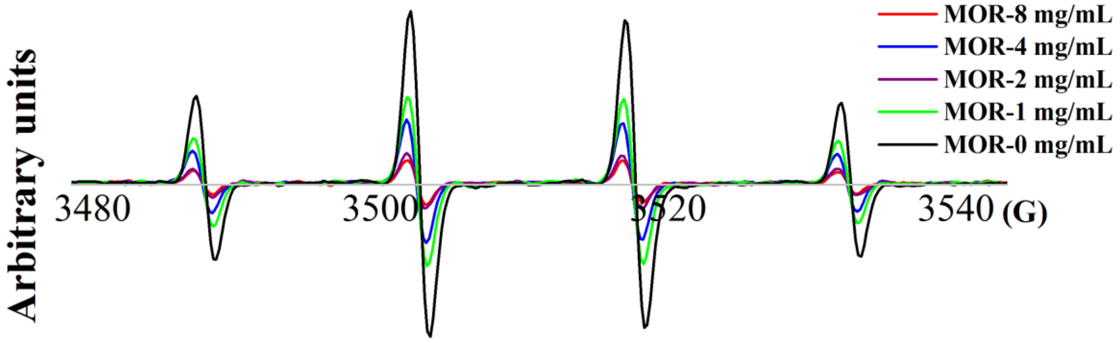

Figure S6. EPR signal strength of HO• at four concentrations of MO-Resin

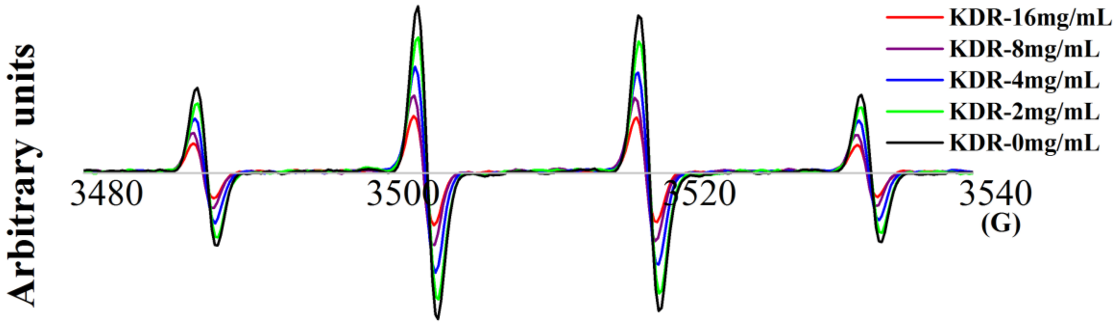

Figure S7. EPR signal strength of HO• at four concentrations of KD-Resin

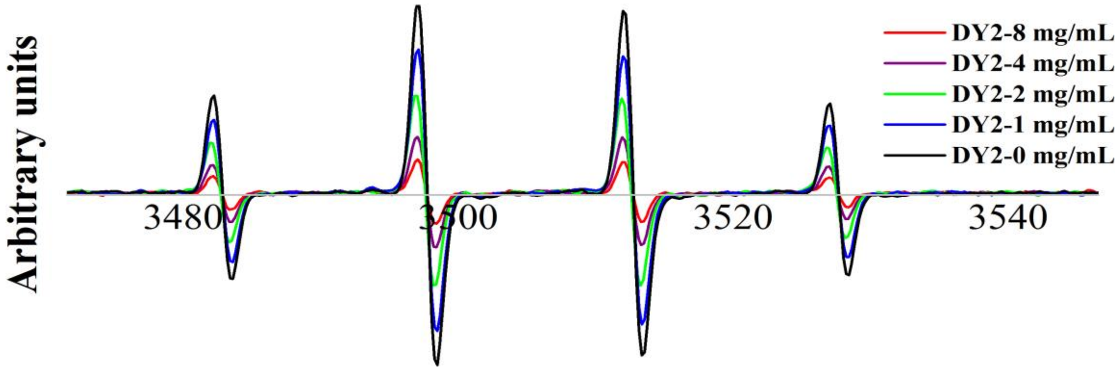

Figure S8. EPR signal strength of HO• at four concentrations of DY-Fraction II

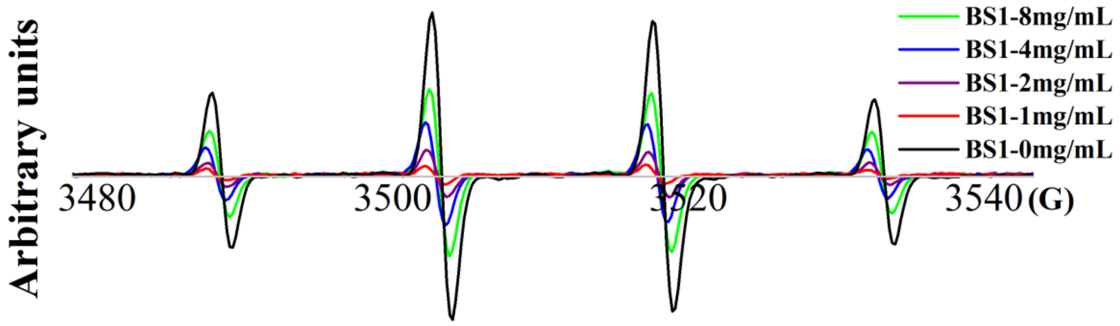

Figure S9. EPR signal strength of HO• at four concentrations of BS-Fraction I

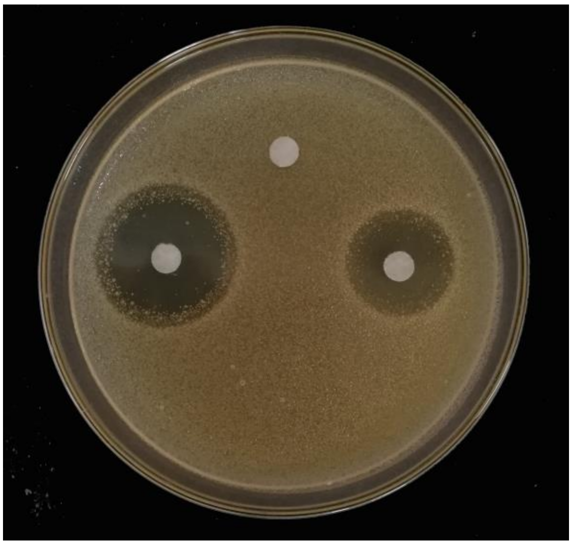

Figure S10. Halo assay of BS-fraction I on *sod1Δ* cells under H_2O_2 stresse

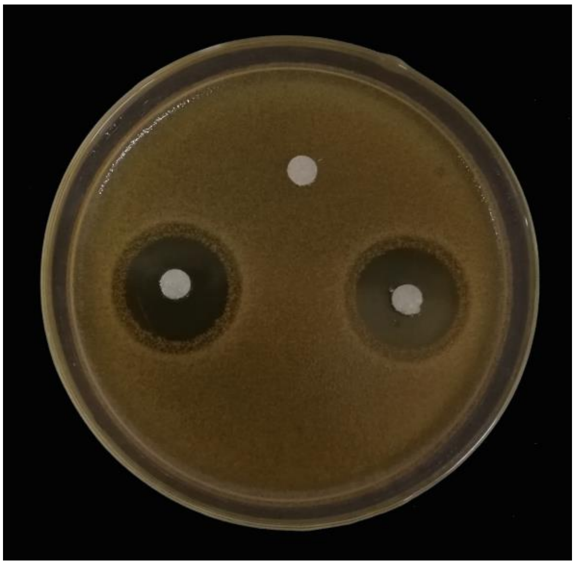

Figure S11. Halo assay of DMSO on *sod1Δ* cells under H_2O_2 stresse

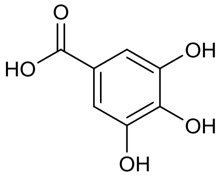

1. 41. Gallic acid

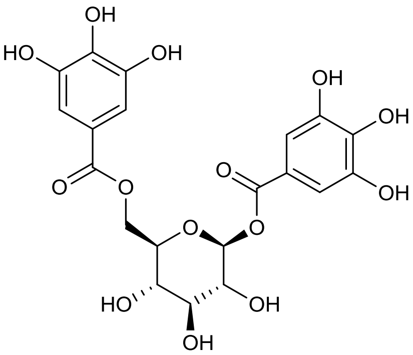

4. 1,6-Digalloyl glucose

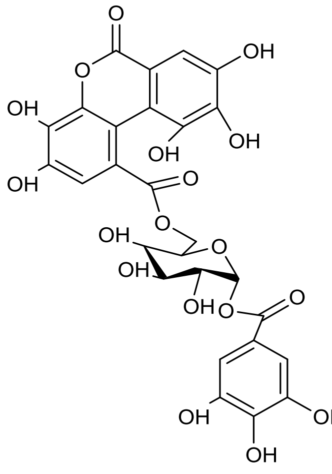

5. 1-O-galloyl-6-O-luteoyl-a-glucose

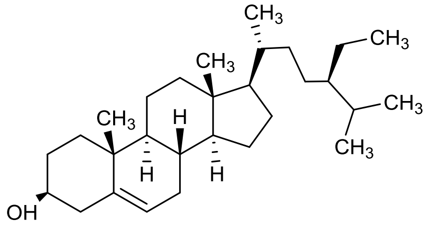

7. β-sitosterol

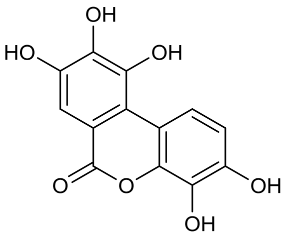

8. 3,4,8,9,10-Pentahydroxydibenzo [b,d]pyran-6-one

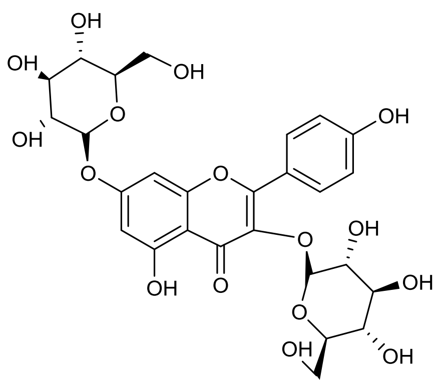

9. Kaempferol-3,7-di-O-β-D-glucopyranoside

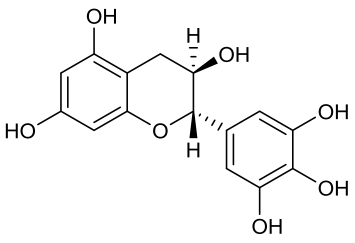

10. 42. 58. (-)-Gallocatechin (GC)

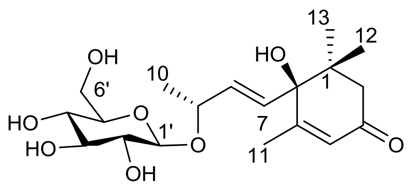

11. (6S,9R)-Roseoside

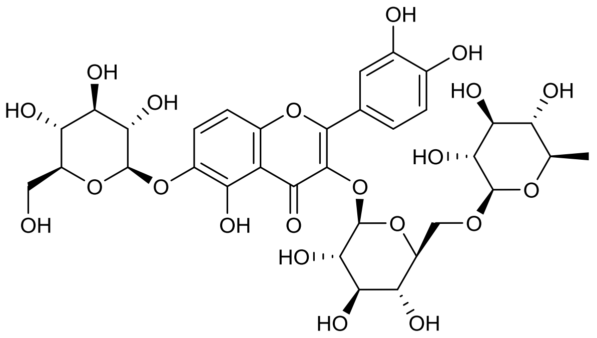

12. 66. Quercetin-3-O-rutinoside-7-O-glucoside

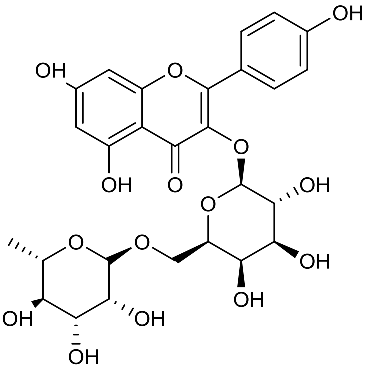

13. Kaempferol-3-O-robinobioside

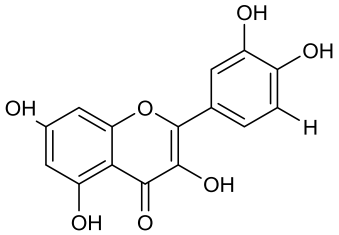

14. Quercetin

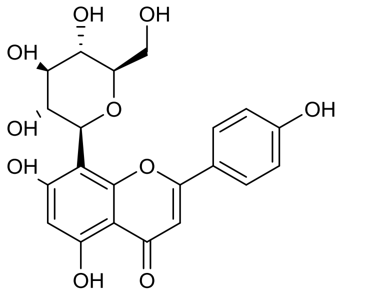

15. Vitexin

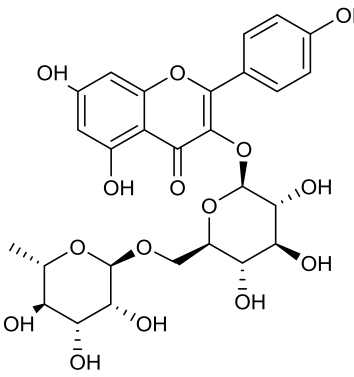

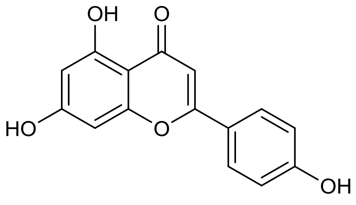

16. 53. 70. Kaempferol-3-O-rutinoside **17. Apigenin**

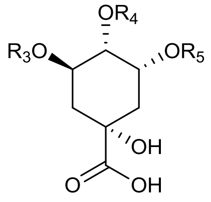

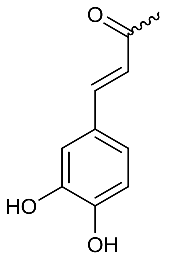

Caffeoyl

22.(43). 23. 24. 31. 32. 33.

	R5	R4	R3
**3-CQA**	**H**	**H**	**Caffeoyl**
**5-CQA**	**Caffeoyl**	**H**	**H**
**4-CQA**	**H**	**Caffeoyl**	**H**
**3,4-diCQA**	**H**	**Caffeoyl**	**Caffeoyl**
**3.5-diCQA**	**Caffeoyl**	**H**	**Caffeoyl**
**4,5-diCQA**	**Caffeoyl**	**Caffeoyl**	**H**

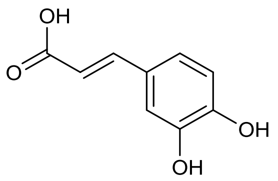

25. Caffeic acid

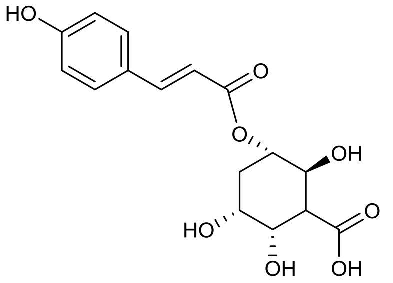

26. 4-O-p-coumaroylquinic acid

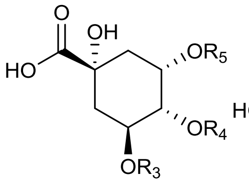

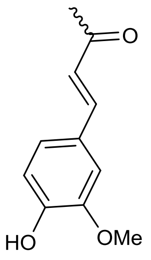

feruloyl

27. 28.

	R3	R4	R5
**3-FQA**	**feruloyl**	**H**	**H**
**5-FQA**	**H**	**H**	**feruloyl**

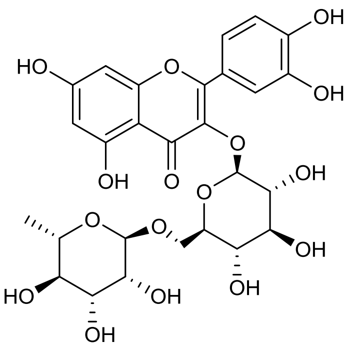

29. Rutin

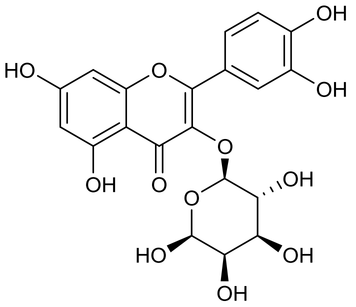

30. 69. Quercetin-3-O-galactoside

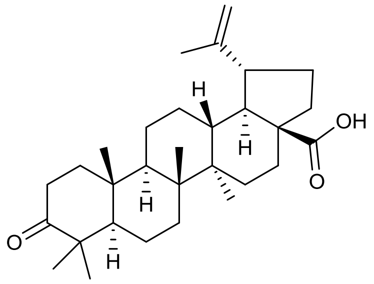

34. Betulonic acid

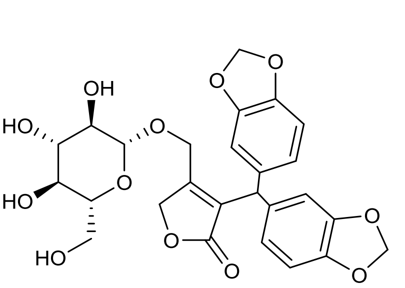

35. Macroanthoin G

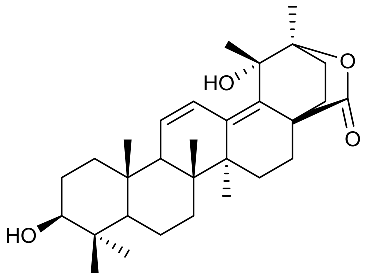

39. α-Kudinlactone

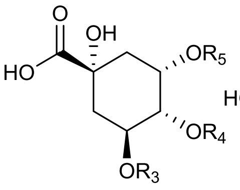

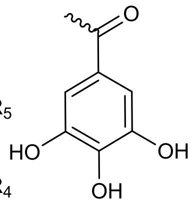

Galloyl

40. (DY) **56. 57.** (BS)

	R3	R4	R5
**5-GQA**	**H**	**H**	**Galloyl**
**3-GQA**	**Galloyl**	**H**	**H**

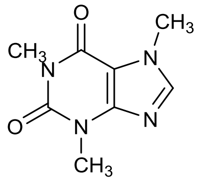

46. 61. Caffeine

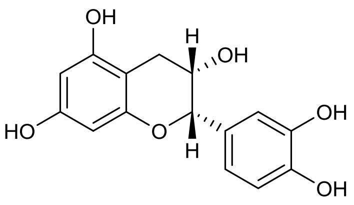

44. 59. (-)-Epigallocatechin (EGC)

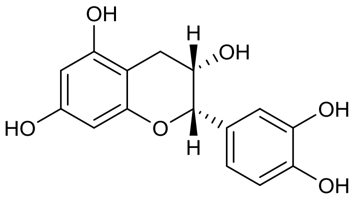

45. 60. (+)-Catechin (C)

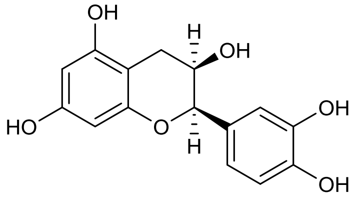

47. (-)-Epicatechin (EC)

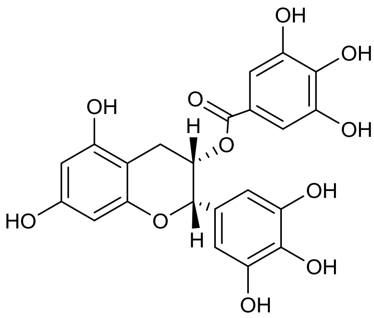

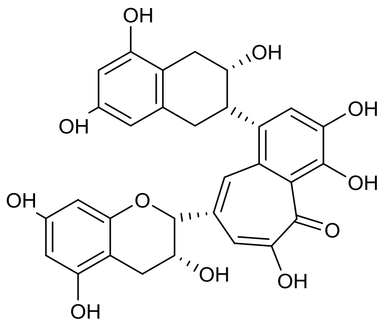

48. 62. (-)-Epigallocatechin-3-gallate (EGCG)

49. 64. Theaflavin

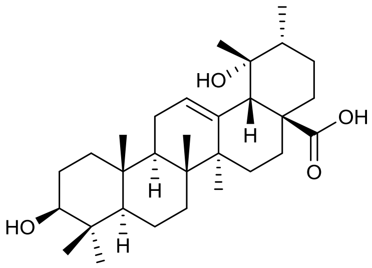

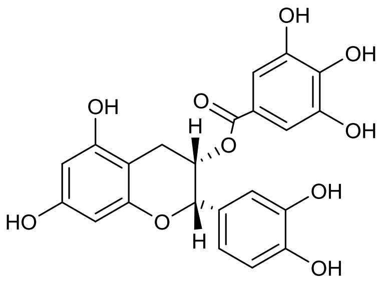

50. Pomolic acid

51. 67. (-)-Epicatechin-3-gallate (ECG)

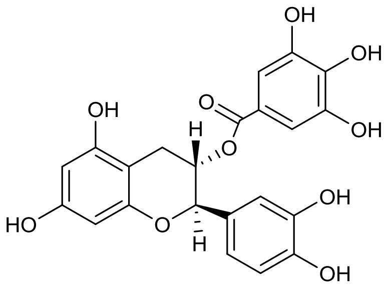

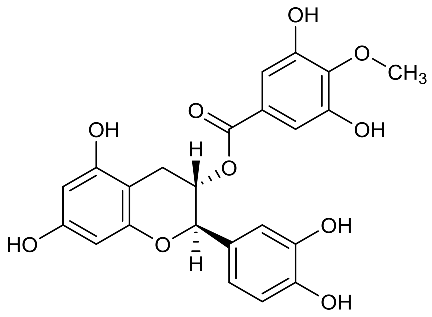

52. 68. (-)-Catechin-3-gallate (CG)

54. (-)-Catechin-3-O-(4-O-methyl) gallate

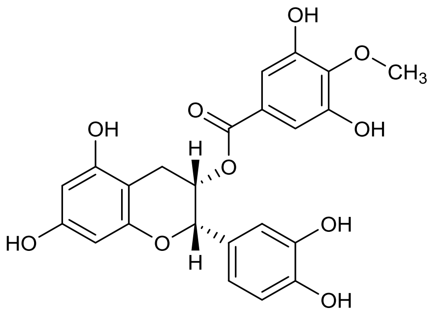

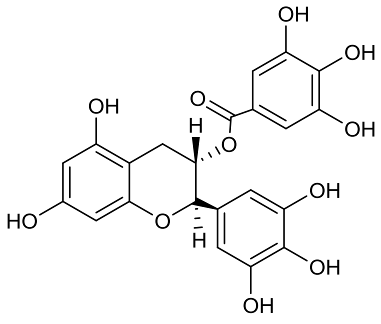

55. (-)-Epicatechin-3-O-(4-O-methyl) gallate

63. (-)-Gallocatechin-3-gallate (GCG)

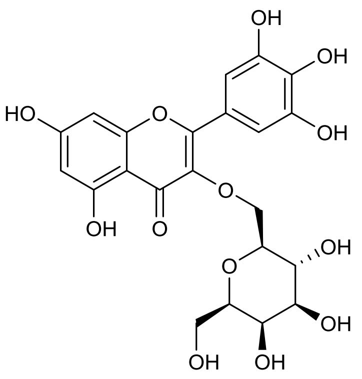

65. Myricetin 3-O-β-L-galactopyranoside

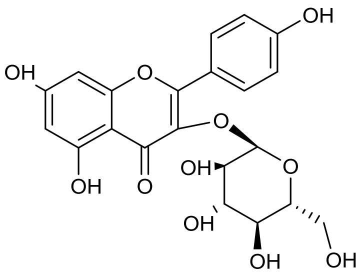

71. Kaempferol-3-O-glucoside

Figure S12. The structures of the main compound of four kinds of teas.
